# Augurio de nostálgica despedida: situación de enfermería bajo la teoría de Swanson*

**DOI:** 10.15649/cuidarte.3088

**Published:** 2023-12-24

**Authors:** Wendy Johana Gómez Domínguez, Helena Guerrero de Caballero

**Affiliations:** 1 . Universidad de Sucre, Sincelejo, Colombia. Estudiante de doctorado en enfermería, Universidad de Antioquia, Medellín, Colombia. Email: wendy.gomez@unisucrevirtual.edu.co Universidad de Antioquia Universidad de Antioquia Medellín Colombia wendy.gomez@unisucrevirtual.edu.co; 2 . Universidad de Sucre, Sincelejo, Colombia. Email: helena.guerrero@unisucre.edu.co Universidad de Sucre Universidad de Sucre Sincelejo Colombia helena.guerrero@unisucre.edu.co

**Keywords:** Atención de Enfermería, Servicio de Cirugía en Hospital, Satisfacción del Paciente, Narrativa Personal, Nursing Care, Surgery Department, Hospital, Patient Satisfaction, Personal Narrative, Cuidados de Enfermagem, Centro Cirúrgico Hospitalar, Satisfago do Paciente, Narrativa Pessoal

## Abstract

**Introducción:**

Se narra una experiencia donde el sujeto de cuidado es una enfermera pionera de la disciplina de la Enfermería en Colombia, quien recibió atención de una egresada y una colega, en donde se vivencia cada momento de la atención y el crecimiento para la persona cuidada y cuidadoras.

**Objetivo:**

Comprender la importancia del cuidado de enfermería para el paciente y la enfermera a través de la narración y análisis de una situación de enfermería.

**Materiales y Métodos:**

El análisis se centra en los conceptos del metaparadigma, la visión del mundo de enfermería, los patrones de conocimiento y la teoría de rango medio de los cuidados de Kristen Swanson, los resultados se visibilizan en la relación enfermera-paciente, en el proceso de enfermería, en la consecución de la integridad, curación, sanación y en la vivencia de la nostálgica despedida de la paciente.

**Conclusión:**

La narración de enfermería es relevante para la teoría, práctica e investigación, porque a través de ella el profesional de enfermería da a conocer eventos que vivencian el quehacer diario y visibiliza los cuidados brindados en interacción con el otro, lo que permite aportar a la evolución del conocimiento de la disciplina.

## Abstract (en)

**Introduction:**

An experience is narrated in which the subject of care is a pioneer nurse in the discipline of nursing in Colombia who was cared for by a graduate and a colleague, and in which each moment of care and growth is experienced for the cared-for person and the caregivers.

**Objective:**

To understand the importance of nursing care to the patient and the nurse by narrating and analyzing a nursing situation.

**Materials and Methods:**

The analysis focuses on the concepts of metaparadigm, nursing worldview, patterns of knowing, and Kristen Swanson's middle-range theory of caring. The results are visible in the nurse-patient relationship, the nursing process, the achievement of wholeness and healing, and the experience of the patient's nostalgic farewell.

**Conclusion:**

Nursing narratives are relevant to theory, practice, and research because, through them, nursing professionals make known events that are experienced in daily work and make visible the care provided in interaction with others, contributing to the evolution of knowledge of the discipline.

## Resumo

**Introdujo:**

Narra-se uma experiencia em que o sujeito do cuidado é uma enfermeira pioneira da disciplina de Enfermagem na Colombia, que recebeu cuidados de uma graduada e de uma colega, onde se vivencia cada momento de cuidado e crescimento da pessoa cuidada e cuidadores.

**Objetivo:**

Compreender a importancia do cuidado de enfermagem para o paciente e para o enfermeiro por meio da narragáo e análise de uma situagáo de enfermagem.

**Materiais e Métodos:**

A análise centra-se nos conceitos do metaparadigma, na visáo de mundo da enfermagem, nos padróes de conhecimento e na teoria do cuidado de médio alcance de Kristen Swanson, os resultados sáo visíveis na relagáo enfermeiro-paciente, no processo de enfermagem, na concretizagáo de integridade, cura e na vivencia da nostálgica despedida do paciente.

**Conclusao:**

A narrativa de enfermagem é relevante para a teoria, a prática e a pesquisa, pois por meio dela o profissional de enfermagem dá a conhecer acontecimentos que vivencia o cotidiano do trabalho e torna visível o cuidado prestado na interagáo com o outro, o que permite contribuir para a evolugáo do conhecimento da disciplina.

## Introducción

“La narrativa es considerada una herramienta pedagógica para el conocimiento de enfermería” ^1^, por medio de ella se pueden contar historias de interacción que tiene el profesional de enfermería con las personas^1^. Es decir, a través de estas, se cuentan situaciones de enfermería, consideradas experiencias vividas entre el paciente y la enfermera, donde ambas partes tienen la oportunidad de crecer en el cuidado.

Más allá de narrar, se invita a pensar, analizar, reflexionar y construir conocimientos, que dan cuenta de la comprensión del cuidado con aportes a la mejora de la práctica de enfermería. La presente narrativa se analizó según los conceptos del metaparadigma, una de las visiones del mundo de enfermería, los patrones de conocimiento enfermero y una teoría de rango medio.

Es necesario distinguir que los conceptos del metaparadigma de enfermería permiten identificar fenómenos centrales de interés para la disciplina^2^. Mientras que las visiones del mundo de enfermería son perspectivas y formas de ver la realidad desde el punto de vista ontológico y epistemológico^3^.

Los patrones de conocimiento en enfermería en sí mismo, proporcionan perspectivas y formas de conocer en la disciplina, permiten dar respuesta a los problemas que surgen en la profesión^4^; por lo tanto, dan autenticidad al cuidado que brinda el profesional enfermero, son considerados esenciales para lograr el dominio de la disciplina, lo que implica los avances en la ciencia de enfermería (patrón empírico). Referente a la capacidad de creatividad e innovación, de empatía y percepción de las necesidades de la persona que se cuida, envuelve la esencia de la realidad en múltiples dimensiones (patrón de conocimiento estético), para lo cual, ser enfermero (a) incluye reconocerse a sí mismo y a los demás como un ser multidimensional que requiere entablar relaciones de reciprocidad (patrón de conocimiento personal), en donde llegar al otro genera salud y bienestar, lo que involucra hacer lo correcto (patrón de conocimiento ético)^5^.

En referencia a las teorías de rango medio, estas permiten abordar aspectos específicos de las situaciones de enfermería y realizar preguntas precisas de la práctica de enfermería, consideradas la evidencia teórica de la aplicabilidad y el resultado^6^. Los conceptos del metaparadigma, las visiones, los patrones de conocimiento y la teoría de rango medio utilizadas en el análisis de la presente narrativa hacen parte de la estructura jerárquica del conocimiento contemporáneo de enfermería^2^. La narrativa expuesta, se analizó gracias a las preguntas orientadoras realizadas por las autoras, entre ellas: ¿De qué forma podemos bajar la abstracción de los conceptos del metaparadigma a la realidad práctica vivida con el sujeto de cuidado?, ¿Cómo se hicieron evidente los patrones de conocimiento en enfermería?, ¿Qué visión de mundo en enfermería se colocó en práctica al brindar cuidado?, ¿Qué teórica de enfermería plasmó el cuidado brindado en la situación narrada?, además, se resalta el papel de las enfermeras en la producción del conocimiento disciplinar, debido al gran rol que tienen en la promoción de la salud y en la reducción de la morbimortalidad de las personas^7^.

## Materiales y Métodos

El material utilizado es una narrativa de la situación de enfermería construida por dos enfermeras desde su experiencia en la práctica como líder de un servicio de cirugía y como colega acompañante, esta narrativa fue almacenada en Mendeley Data^8^, el método utilizado fue el enfoque de análisis de la narrativa en relación con los conceptos del metaparadigma, visión del mundo, patrones de conocimiento y teoría de rango medio en enfermería.

### Narrativa

### Augurio de nostálgica despedida: situación de enfermería bajo la teoría de Swanson

Haciendo memoria a mi primer día de clases de pregrado en la sede de una universidad, en donde se ubica el Programa de Enfermería de la Región, ahí estaba la protagonista de esta narrativa, “Yeisi” (nombre ficticio). La recordamos como una mujer robusta, de piel morena, con cabellera menuda, ojos grandes que irradiaban una mirada intensa llena de vigor, sus labios rojos siempre mostraban pequeños tics de muecas graciosas, su cuello y orejas siempre ocupadas por sencillos y extraños accesorios artesanales que confluían con vestidos elegantes de múltiples modas sin límites para la edad y el tiempo, también lucía pantalones que encajaban perfecto en tan voluptuosas caderas y fuertes piernas que la acompañaron en todo su caminar, cualidades que hacían notoria su presencia e imponía autoridad y alegría, propia de su estirpe morena.

Yeisi fue un ícono para estudiantes y docentes, su sensual amabilidad y sencillez incitaba al diálogo con ella, dada su alta capacidad en la resolución de problemas, lo que permitía a menudo obtener buenos logros, y ante todo, le apasionaba la dirección del talento humano en enfermería, la investigación e innovación que la llevó a direccionar grandes proyectos de orden nacional e internacional, logrando plasmar un liderazgo en la disciplina y proponer programas de posgrados en donde tuvimos la oportunidad de ser egresadas y docentes con excelentes implicaciones en la vida laboral y personal.

Meses después, el 21 de abril del año 2016, mientras me encontraba trabajando en el turno de la tarde en el servicio de Cirugía de una clínica de la ciudad, ingresó Yeisi como la identificamos, en el rol de paciente, a realizarse una osteosíntesis de radio derecho, cuando la observé, sentí alegría de poder atenderla con prácticas de cuidado seguro, lo que infirió en ella un desborde de emociones que mostraban sentimientos de agradabilidad y de inmediato hizo muecas de felicidad en su rostro como solía hacerlas.

Al mismo tiempo mostró una ligera preocupación al preguntar: ¿Será muy dolorosa la cirugía? a lo que respondí que durante el acto quirúrgico no sentiría dolor, además, se le dio orientación en el servicio, en la vestimenta que debía llevar y se procedió a la preparación prequirúrgica con posterior atención en el transoperatorio (donde se le explicaron los procedimientos a realizar, la administración de la profilaxis antibiótica, analgésicos indicados, cuidados mientras estaba bajo anestesia general, en compañía del resaltado equipo quirúrgico) y sala de recuperación, además, fue asistida por una de sus colegas que la acompañaba, quien le ofreció atención en el manejo del dolor, asistencia en el dominio de sus emociones con apoyo espiritual, vigilamos su despertar, se le probó la vía oral, la cual fue bien tolerada, y recuperada de su estado posoperatorio inmediato se le procedió a explicar y entregar un plan de cuidados para el hogar.

Ahora bien, me evoca el recuerdo de un turno tranquilo, silencioso y aforo mínimo que permitió interactuar directamente en el cuidado de enfermería, contribuyendo así a una cirugía exitosa. En la de alta, dado que era una cirugía ambulatoria, se ubicó en la silla de ruedas en compañía de su familiar y una de sus colegas que le atendía de manera permanente, Yeisi bajo una agradable sonrisa en su rostro expresó: ¡Gracias a todos!

Cuatro meses después, nos ofreció la bienvenida en una solemne ceremonia de inicio de un programa de posgrado (maestría en enfermería) y en febrero de 2017 en convocatoria a docentes catedráticos fui favorecida y me permitió ser docente, cargo que aún conservo por mi alta vocación y sentido de pertenencia con la profesión, en donde sigo aprendiendo y enseñando de tan hermosa disciplina.

En la memoria de mis recuerdos, yace una fecha inolvidable.. .Era un 12 de mayo del año 2019 cuando celebrábamos el día de la Enfermería, y Yeisi inauguró esta fecha conmemorable frente a una multitud de enfermeras y estudiantes, donde expresó en su discurso: “Me siento agradecida con Dios por ser Enfermera, esto ha contribuido a mi crecimiento personal y profesional, me dio base en el acervo de conocimientos de enfermería que requería para los últimos momentos de vida de mi madre”, no obstante, agregó: “Por favor, nosotros somos enfermeras y enfermeros que llevamos el cuidado con calidad por nuestras venas y nos permite entregarle con amor hasta una sábana para proteger del frío a nuestro sujeto de atención, a desplegar en ellos la razón de ser del cuidado que nos hace más humanos”.

Estuvimos tan atenta a sus palabras, sin perder de vista sus expresiones, las cuales marcaron bellos comentarios, que auguraban ser últimas intervenciones como líder de enfermería en una fecha tan especial, sentimiento nostálgico mezclado con sus mágicas palabras que cultivaban en cada una de nosotras un imaginario creativo para sembrar en nuestra mente y alma cuidados de enfermería transformadores que parten de la cotidianidad. Meses después, hizo presencia la pandemia por Covid-19, Yeisi se jubiló.Pero, ¡Que pesadilla!,¡Que tragedia!, que rato tan amargo vivimos la tarde del 2 de agosto del año 2020, cuando expiró la conocida, admirada y distinguida Yeisi, solo se escuchaba: ¡Ha muerto!, ¡Ha muerto!, ¿Por qué ella Dios mío?, todo parecía un sueño del que no nos despertábamos, ¡Si!, definitivamente había muerto una exponente con grandes perspectivas de proyectos. y lo peor fue saber que la pandemia Covid-19 presente en la humanidad se la había llevado de la vida terrenal.

El consuelo se halló en las oraciones que brindaban fortaleza en el más allá, era como algo irreal que no permitió darle su último adiós funeral porque su cuerpo fue incinerado por la bioseguridad reinante del momento. Que partida tan injusta, triste y reflexiva, en donde el equilibrio de la evidencia y uso de la experiencia del duelo formaron una simbiosis contradictoria en la aceptación de su partida, sin embargo, consuela que ella probablemente está gozando de la presencia de Dios y vivirá para siempre en nuestra memoria una gran mujer, madre, maestra, esposa, amiga, compañera, profesora, ama de casa, y excelente enfermera.

En esta situación de enfermería que se narra se visibilizan significados en la intuición del ser como enfermera cargada de sentimientos y emociones que trascienden barreras del fenómeno de la vida y la muerte, lleva como enseñanza, la alegría que la profesión de Enfermería refleja como arte y ciencia del cuidado, es decir, el disfrute de cada momento vivido, el estar motivado a brindar cuidado y ofrecer bajo diversas circunstancias atención humanizada. Se hacen presente las voces del cuidado en una situación de enfermería y a la vez se evidencia el augurio de la nostálgica despedida de una gran mujer.

Gracias por ayudarnos a crecer, por ser parte de nuestros espejos, nunca la olvidaremos porque será para nosotras una enfermera insepultable en nuestra memoria. “Se sepulta el cuerpo, se sepultan las cenizas, pero las grandes personas, sus buenos recuerdos vivirán para siempre”. Gracias por ser autora de la historia de enfermería en el universo.

### Análisis de los conceptos del metaparadigma

*Definición de los Conceptos del Metaparadigma de Enfermería en la situación.* Estos conceptos se consideran el primer componente de la estructura del conocimiento contemporáneo en enfermería y permiten identificar el fenómeno central de interés para la disciplina. Entre ellos: Persona, ambiente, salud y enfermería^2^.

- Persona: “Hace referencia a los individuos, familias, comunidades y grupos que son participantes en enfermería”^(2)^. En la situación narrada esa persona es Yeisi, exactamente *“Una mujer robusta, de piel morena, con cabellera menuda, ojos grandes..."* además, una mujer con morbilidad y sentimientos de alegría, ansiedad e incertidumbre.
- Ambiente: “Son las personas significativas para el individuo, el hogar, los escenarios como clínicas, hospitales, la sociedad como un todo donde las acciones de enfermería tienen un espacio importante”^2^, es decir, el entorno del paciente y de enfermería^9^. El ambiente es la clínica y exactamente el servicio de cirugía, por otra parte, el encuentro significativo de la maestra con su egresada y el acompañamiento de su colega. Es evidente cuando se vive el momento inicial de paciente-enfermera *“Mientras me encontraba trabajando en el turno de la tarde en el servicio de Cirugía de una clínica de la ciudad, ingresó Yeisi como la identificamos, en el rol de paciente".*
- Salud: “Estado de bienestar en el momento en que se brindan los cuidados de enfermería”.^2^^)^ Se resalta el estado de salud inicial al ingreso de la paciente, llegó estable con una cirugía programada, pero cursaba con una morbilidad que tal vez limitaba el ejercicio de su rol diario, con los cuidados y la intervención se pudo conseguir la solución y sanación del problema de salud, así como la satisfacción de la paciente. En la narrativa se puede notar cuando se plasma: *“Me evoca el recuerdo de un turno tranquilo, silencioso y aforo mínimo que permitió interactuar directamente en el cuidado de enfermería, contribuyendo así a una cirugía exitosa".*
- Enfermería: Se refiere a las acciones de enfermería como un proceso sistemático de valoración, diagnóstico, planeación, intervención y evaluación. También a las metas y resultados de las acciones de enfermería^2^. En la narrativa enfermería estuvo presente desde el ingreso hasta el egreso de la paciente del servicio de cirugía, lo cual deja claro que cada una de las áreas del servicio requieren del actuar y guía del profesional enfermero, debido a que en el ingreso (preparación de pacientes) se pudo valorar a la paciente, realizar diagnósticos con el fin de planear cuidados coherentes con la realidad, esto es indispensable para intervenir (quirófano - recuperación) y por último evaluar el cuidado brindado, que en este caso fue muy exitoso (egreso). Se evidencia en varios momentos de la narrativa, aquí se menciona uno de ellos: *“Se le dio orientación en el servicio, en la vestimenta que debía llevar.".*

### Análisis de la visión

*Visión del mundo de Enfermería en la situación.* En la narrativa se hace evidente la visión interactiva integrativa, la cual toma su origen desde el postpositivismo, le impregna gran relevancia al ser humano considerándolo un ser holístico (la enfermera no solo se quedó con la necesidad física de la paciente y su limitación, ella observó más allá, notó los sentimientos, necesidades emocionales y espirituales).

El ambiente es multidimensional (el ambiente donde estaba la paciente a pesar de tener gran relación con su profesión no era su ambiente natural, se trataba de una clínica con personal extraño y en el rol de paciente se tienen expectativas y sentimientos al ingresar a un servicio de cirugía), en cuanto a la salud es dinámica con cambios recíprocos (la persona ingresó con un diagnóstico prequirúrgico el cual se modificó una vez terminó la intervención quirúrgica), además, con los cuidados de enfermería y la intervención interdisciplinaria la paciente mejoró su situación de salud a través de cuidados seguros acorde a sus necesidades reales. Enfermería es idóneo en el captar lo que el otro necesita, analiza la situación de salud como base del cuidado y mejora de la calidad de vida de esa persona que busca su bienestar^3^.

### Análisis de los patrones de conocimiento

*Patrones de conocimiento en Enfermería en la situación.* Los patrones son conjunto de conocimientos y las formas de conocer en Enfermería, le dan fundamentación a la práctica, permiten mejorar la enseñanza y aprendizaje de la disciplina de enfermería. Barbara Carper identificó cuatro patrones fundamentales de conocimiento en enfermería como: Patrón de conocimiento empírico (ciencia de enfermería), estético (arte de enfermería), personal y ético^5^.

- Patrón de conocimiento empírico (ciencia de enfermería): Se refiere a los conocimientos empíricos propios de la disciplina de enfermería, fueron los años cincuenta, los inicios de la ciencia de enfermería, gracias a las necesidades críticas de organizar sistemáticamente el conocimiento enfermero en leyes, modelos y teorías que permitieron describir, explicar y predecir fenómenos de interés de la disciplina. Fue el primer patrón fundamental^5^. En la narrativa este patrón se evidencia cuando se hace mención a:

*“Se le dio orientación en el servicio, en la vestimenta que debía llevar y se procedió a la preparación prequirúrgica”.*

*"Se le explicaron los procedimientos a realizar, la administración de la profilaxis antibiótico...” “Me siento agradecida con Dios por ser Enfermera, esto ha contribuido a mi crecimiento personal y profesional.”.*

En la narrativa, este patrón llevó a establecer la importancia de tener claro los conocimientos conexos con las prácticas que permite brindar atención al paciente quirúrgico.

- Patrón de conocimiento estético (arte de enfermería): Es expresivo, implica conocimiento subjetivo y comprensión, es un arte creativo, donde enfermería es capaz de crear e innovar en las diferentes formas de ayudar a los demás. Incluye reconocimiento y percepción. Además, la empatía es una modalidad esencial de este patrón^5^. Se hace evidente en los siguientes momentos:

*"Cuando la observé, sentí alegría de poder atenderla con prácticas de cuidado seguro.”.*

*"Por favor, nosotros somos enfermeras y enfermeros que llevamos el cuidado con calidad por nuestras venas.”.*

Por consiguiente, este patrón también es importante y hermoso para la disciplina, es todo un arte porque no todos los profesionales pueden llegar a las personas como lo hace enfermería.

- Patrón de conocimiento personal: Considerado el patrón de conocimiento que genera problemática, debido a que es difícil de enseñar y dominar. Es el encuentro y esfuerzo que se hace por conocerse a sí mismo. Es esencial para comprender la salud con relación al bienestar individual. Es subjetivo y niega toda orientación impersonal^5^. Se presencia al expresar:

*"Ahí estaba la protagonista de esta narrativa, Yeisi”. "Su sensual amabilidad y sencillez incitaba al diálogo con ella, dada su alta capacidad en la resolución de problemas .”. "Me permitió ser docente, cargo que aún conservo por mi alta vocación”."Pero ¡Que pesadilla!¡Que tragedia!, que rato tan amargo vivimos la tarde del 2 de agosto del año 2020, cuando expiró la conocida, admirada y distinguida Yeisi, solo se escuchaba: ¡Ha muerto!, ¡Ha muerto!”.*

El patrón que aquí se menciona permitió que la enfermera recordara su misión y su compromiso social siempre en beneficio de los demás. Además, este patrón embarga reflexión y conexión para descubrirse a sí mismo y al otro, no considera imponer creencias^10^.

- Patrón de conocimiento ético: Considera la enfermería como un servicio social valioso, con el fin de conservar la vida, aliviar el sufrimiento y mejorar la calidad de vivir, entonces traspasa el cumplimiento de códigos éticos de enfermería, debido a que hace referencia a las acciones voluntarias sujetas al juicio de lo correcto e incorrecto del profesional de enfermería^5^. Se hace evidente cuando se menciona:

*“Vigilamos su despertar, se le probó la vía oral, la cual fue bien tolerada...".*

Entonces, este patrón permitió dar a conocer que con todos los pacientes hay que actuar de la mejor forma posible, donde prime la ética, ese deber ser, sin discriminar, con seguridad se puede afirmar que para la enfermera todos los pacientes tienen el mismo valor.

Asimismo, con el pasar del tiempo surgieron otros patrones de conocimiento en enfermería. Estos son:

- Patrón de conocimiento sociopolítico: Garantiza el avance de la disciplina en el sentido social, tiene presente el contexto de enfermería (persona, colectivo, familiar, regional, nacional, entre otros). Abarca el contexto del profesional, paciente y el de la práctica profesional. Este patrón requiere que el profesional de enfermería cuente con liderazgo y habilidades en la comunicación. Fue propuesto por Jill White^4^. Se resalta en la narrativa cuando se expresan:

*“Me evoca el recuerdo de un turno tranquilo, silencioso y aforo mínimo que permitió interactuar directamente en el cuidado de enfermería".“Era un 12 de mayo de 2019 cuando celebrábamos el día de la Enfermería, y Yeisi inauguró esta fecha conmemorable frente a una multitud de enfermeras y estudiantes".*

Debido a lo anterior, se puede mencionar que las cuidadoras de Yeisi valoraron el contexto en cada momento del cuidado, asimismo, en la narrativa la paciente fue una gran profesional reconocida para la sociedad y a la vez ella aportó mucho al desarrollo social.

- Patrón de conocimiento emancipatorio: Persigue lograr la justicia y equidad de los servicios de salud y de enfermería, también valora el contexto para construir relaciones equitativas. Fue propuesto por Peggy Chinn y Maeona Kramer^4^ .Se hace evidente cuando se narra lo siguiente:

*“Que partida tan injusta, triste y reflexiva". “Gracias por ayudarnos a crecer, por ser parte de nuestros espejos, nunca la olvidaremos porque será para nosotras una enfermera insepultable en nuestra memoria". “Logró proponer posgrados en enfermería".*

En la narrativa este patrón se hace presente porque la enfermera actuó de forma justa en todo momento, además, las enfermeras agradecieron por el crecimiento que dejó Yeisi en muchas personas, por otra parte, llegaron a considerar la partida de Yeisi como una partida injusta en el ámbito personal y de enfermería, también lograron percibir que la paciente era una persona que cada día luchaba por mejorar los servicios de salud y la formación de enfermería.

• Patrón de conocimiento espiritual: Promueve el bienestar universal, un patrón distinto de los demás, es fundamental para la estructura y enfoque de la disciplina de enfermería, además, permite ver al ser humano en su totalidad y orientar la práctica enfermera, considerado una cualidad del ser humano que proporciona significado y propósito en la vida. Es de resaltar, que la espiritualidad ha estado presente en la historia de la enfermería porque la naturaleza espiritual de los seres humanos implica que enfermería se unte de conocimiento espiritual. Por lo tanto, este patrón se relaciona con el metaparadigma de la disciplina^11^. Se evidencia cuando: *“El consuelo se halló en las oraciones que brindaban fortaleza en el más allá, era como algo irreal que no permitió darle su último adiós..."*

En la narrativa se evidenció, cuando la enfermera fue capaz de conectarse consigo misma y con la paciente, lo que le permitió ver más allá de lo tangible.

### Teoría de Kristen Swanson en la situación de enfermería

La situación narrada se relaciona con la teoría de los cuidados de Swanson; esta permitió la comprensión de la situación, al dar relevancia a la interacción y presencia de la enfermera con la paciente, lo que condujo a colocar en práctica el proceso de enfermería, y conseguir la integridad, sanación y lograr vivenciar la nostálgica despedida de Yeisi, bajo el compromiso y la responsabilidad que implica brindar cuidado; asimismo, permitió converger lo histórico, lo antropológico, académico y lo personal en la relación del cuidado^12^.

Swanson con su teoría definió el concepto de cuidado como “Una manera de relacionarse con otro” ^13^^)^ y describió cinco procesos básicos del cuidado, como:

- Saber: Implica esforzarse por comprender el evento que vive el otro, el significado que tiene para él, evitar suposiciones y comprometerse con el cuidado^13^. En la narrativa este proceso básico del cuidado se llevó a cabo gracias a la presencia de la enfermera con su paciente, en este momento la enfermera conoció que la persona consultaba por una cirugía programada, además, se logró comprender la ansiedad e incertidumbre reflejada en la paciente por la intervención quirúrgica, esto permitió planear el cuidado a brindar.
- Estar con: Es estar emocional y físicamente con la persona que se cuida, estar disponible, compartir sentimientos, sin perturbar al sujeto de cuidado^13^. El proceso de “Estar con el sujeto de cuidado” en la situación de enfermería se presenció desde el ingreso hasta el egreso de la paciente, se traduce en esa escucha activa de la enfermera.
- Hacer para: Es hacer por el otro lo que la misma persona haría para cuidarse, incluye anticipar las necesidades, brindar cuidado de manera competente, hábil, proteger al otro, preservar su dignidad^13^. La enfermera hizo varias intervenciones y actividades con el fin de contribuir a la salud, calidad de vida y satisfacción del paciente, aplicó el proceso de atención de enfermería, fue amable y empática.
- Habilitar: Es facilitar el paso de la otra persona, concibe explicar, apoyar, permitir, validar sentimientos, pensar las cosas y retroalimentar^13^. Se observó el proceso de “Habilitar” cuando la enfermera permitió que su paciente se expresara, también a través de la observación porque llevó a percibir los sentimientos.
- Mantener la creencia: Consiste en mantener la fe en la capacidad del otro para superar una situación, ayudarlo a encontrar sentido, tenerle alta estima y concebir la esperanza en el evento que se vive para llegar a un futuro con significado^13^. En la situación narrada la enfermera siempre respetó las creencias de la persona a la cual le brindó cuidado, se evidenció desde la alta estima que tuvo con la paciente.

## Conclusiones

La narración de las situaciones de enfermería es relevante para la teoría, práctica e investigación, porque a través de ellas pueden darse a conocer eventos que se viven en el quehacer diario y muchas veces resultan invisibles, pero, sin los cuidados brindados en cada experiencia con el otro, no sería posible la evolución del conocimiento de la disciplina.

Las situaciones de enfermería se convierten en un tipo específico de investigación, porque a través de ellas se produce conocimiento a partir de la interacción humana, lo que fortalece la ciencia de enfermería y por lo tanto la mejora de la práctica.

Se cuenta lo que se vive con pasión, sentimiento, amor, tristeza, dolor, alegría y que permite crecer al ser humano, como lo fue la situación *“Augurio de nostálgica despedida: situación de enfermería bajo la teoría de Swanson”.*
